# Roadmap of Flexible Electrodes for Next-Generation Wearable Electronics

**DOI:** 10.1021/acs.energyfuels.5c05463

**Published:** 2026-01-22

**Authors:** Kaaviah Manoharan, Martin Pumera

**Affiliations:** † Future Energy and Innovation Laboratory, Central European Institute of Technology, 48274 Brno University of Technology, Purkyňova 123, Brno 61200, Czech Republic; ‡ Faculty of Electrical Engineering and Computer Science, VSB - Technical University of Ostrava, 17. Listopadu 2172/15, Ostrava 70800, Czech Republic; § Department of Chemical and Biomolecular Engineering, Yonsei University, 50 Yonsei-Ro, Seodaemun-Gu, Seoul 03722, South Korea; ∥ Energy Research institute@ntu (ERI@N), Research Techno Plaza, X-Frontier Block, Level 5, 50 Nanyang Drive, Singapore 637553, Singapore

## Abstract

With the rapid growth of smart innovations, flexible electronics recognized for their lightweight, tremendous flexibility, and extraordinary scalability are growing more integrated into our daily life. Flexible electronics, known for their lightweight, high flexibility, and seamless integration with biological and nonbiological systems, are driving advances in wearable and implantable devices. Central to this progress are flexible electrodes, which enable energy storage, sensing, and health monitoring. This review highlights the evolution of flexible electrode materials over the past decade, focusing on carbon-based systems, transition metal compounds, MXenes, conductive polymers, and metal–organic frameworks (MOFs). Advances in flexible electrolytes, including aqueous, nonaqueous, ionic, and redox gel systems, are also discussed. Key applications span health monitoring, robotics, and plant wearables. We critically analyze material advantages, fabrication challenges, and integration hurdles while outlining future prospects for scalable, biocompatible, and multifunctional electrode systems.

## Graphical abstract

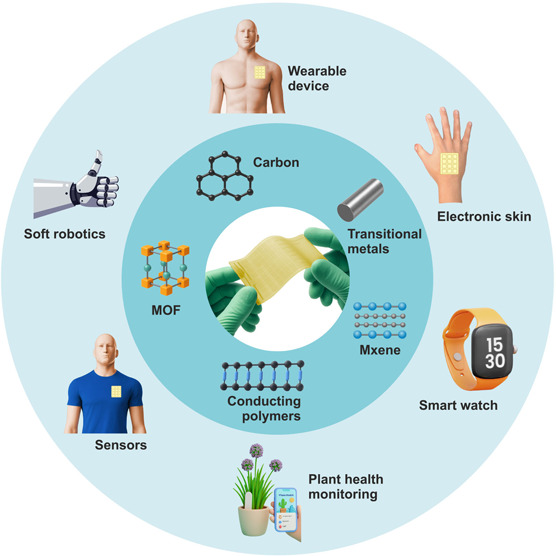

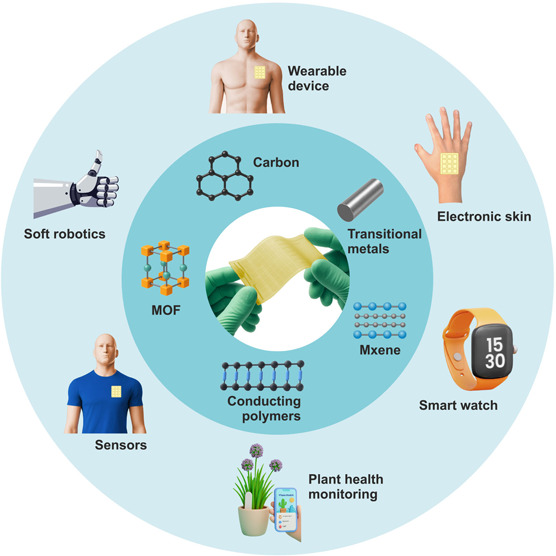

## 1 Introduction

One new technology that makes it possible to produce organic or inorganic materials on pliable and ductile surfaces is flexible electronics. Flexible electronics are different from standard electronics in that they can stretch, compress, bend, fold, and conform to different shapes while still being stable, dependable, and integrated. Because of their versatility, they are ideal for a wide range of environmental circumstances. Flexible electronic devices have recently obtained outstanding ductility and deformation resistance, notably in applications such as wearable healthcare, energy storage, smart electronic skin, and human–computer interface.^1–3^ Because versatile electrodes are required to provide consistent and continuous signal recording, their development has played a significant role in the advancement of this technology. Nevertheless, there are still difficulties in developing flexible electronics with excellent biocompatibility, high toughness, and lightweight characteristics. Since electrodes have a major effect on performance, improving the capabilities of flexible electronics depends on their thoughtful design and manufacture. For a variety of purposes, such as integrating into different surfaces or facilitating folding and rolling for next-generation electronics, flexible electrode materials need to have particular topologies and characteristics. However, in order to be useful, these materials must be functionally reliable. Advances in nanomanufacturing have tremendously accelerated the development of flexible electrodes. Historically, their manufacturing relied on conductive flexible substrates such as hydrogels/aerogels, polymer films, and conductive fibers/fabrics. These standalone electrodes have properties including conductivity, porosity, and flexibility, which make them appropriate for independent use in flexible devices without the need for other functional materials.^4^ Particularly in healthcare applications, their ease of preparation, scalability, abundance of material sources, affordability, and environmental friendliness have attracted a lot of attention. Flexible electrodes nonetheless have drawbacks despite these advantages, such as bulkiness that makes them difficult to integrate and transport, low mechanical strength that limits their use, and poor conductivity that results in erratic signal transmission.^5^

Flexible electrodes made of composite materials with nano functional components aim to provide excellent interface contact and reliable signal transmission. These electrodes are made by layer-by-layer integrating functional components such metal oxides, carbon, graphene, MXenes, and MOFs with a variety of flexible substrates to ensure improved performance and durability.^6–8^ The primary advantage of these electrodes is their ability to allow unlimited carrier transport at the interface, resulting in increased conductivity and more reliable, faster signal transmission. However, they confront challenges with repeatability since repetitive folding and bending and cause the material to disruption or detach from the substrate.^9,10^ This alters the electric field distribution, leading to signal interruptions and obstructing electron and ion transport, ultimately shortening the lifespan of flexible electronic devices.

Flexible electrodes often fail due to fundamental material and interface limitations. Interfacial stress mismatch between rigid active layers and soft substrates generates localized strain concentrations, leading to cracking and gradual delamination during repeated bending. Many electrode systems, particularly transition-metal compounds and conductive polymers, also possess intrinsic brittleness, making them prone to fracture under tensile deformation. In addition, materials such as MXenes and metal oxides exhibit chemical and oxidative instability, which accelerates structural degradation and reduces conductivity under ambient humidity and oxygen exposure. To overcome these limitations, recent advances focus on developing self-healing materials that restore conductivity after damage, designing stress-buffering architectures such as serpentine, wrinkled, or kirigami structures to dissipate mechanical strain, and employing encapsulation or surface-engineering strategies to suppress oxidation and enhance environmental durability. These approaches collectively improve the mechanical robustness and long-term reliability of flexible electrodes.

Additionally, the fabrication process is complex and requires expensive equipment, making large-scale production difficult. As a result of continuous technical development and in-depth research, new flexible electrode designs and fabrication techniques are appearing.^11^ It is becoming more and more possible to create electrodes that are extremely flexible, high-performing, and long-lasting thanks to innovative electronic materials and user-friendly gadgets. Researchers seek to obtain higher electrode performance and stability by the strategic use of flexible substrates, functional materials, and optimum production procedures. Therefore, the risk of detachment can be decreased by carefully choosing suitable electrode preparation techniques that will improve the adherence between the flexible substrate and the functional material. This provides some cost benefits and results in more reliable signal collecting. Overall, despite current obstacles, flexible electrodes continue to be a major area of interest in the development of future electronics, offering a wide range of possible applications. This paper offers a thorough summary of the technical developments in flexible electrodes, including active materials, flexible substrates, production techniques, and new applications.^12,13^ We investigated flexible electrode materials, which have been extensively researched in recent years, from two viewpoints. Growth of electrode materials (metals, carbon-derived materials, MXenes, MOFs, and conducting polymers) and flexible electrolytes. We also discuss recent advances in flexible electrode materials for high-demand areas such as wearable monitoring of health, automation, and plant sensing.

Although several reviews covering flexible electrodes have been published, most of them focus on isolated aspects such as individual material classes, specific sensing platforms, or a single application domain. However, a comprehensive and integrated discussion that simultaneously encompasses flexible electrode materials, flexible electrolytes, fabrication techniques, interfacial/mechanical failure mechanisms, and cross-domain applications remains missing. Existing reviews rarely address emerging challenges such as environmental instability, interfacial stress failure, scalability of manufacturing, coupling in self-powered systems, and multifunctional integration for human, robotic, and plant interfaces. Given the rapid expansion of wearable and biointegrated electronics, a timely and holistic review bridging materials chemistry, device engineering, and real-world application needs is essential. This review aims to fill this gap by providing a unified, up-to-date, and cross-disciplinary overview of flexible electrodes and electrolytes, highlighting both technological limitations and forward-looking opportunities for next-generation wearable platforms.

Figure 1 summarizes the fundamental components and application domains of flexible electrodes. It illustrates how nanomaterials such as graphene, CNTs, and porous carbons impart high conductivity, biocompatibility, and mechanical deformability when integrated with flexible substrates. These material–substrate combinations enable diverse applications, including wearable electronics, electronic skin, and plant-health monitoring. The schematic also highlights key challenges—mechanical reliability, stable signal transmission, long-term biocompatibility, and scalable manufacturing. In parallel, it reflects emerging trends such as energy harvesting and AI/IoT-enabled smart sensing. Together, the figure provides a concise visual framework for the material strategies and technological needs discussed throughout this review.

**Figure 1:**
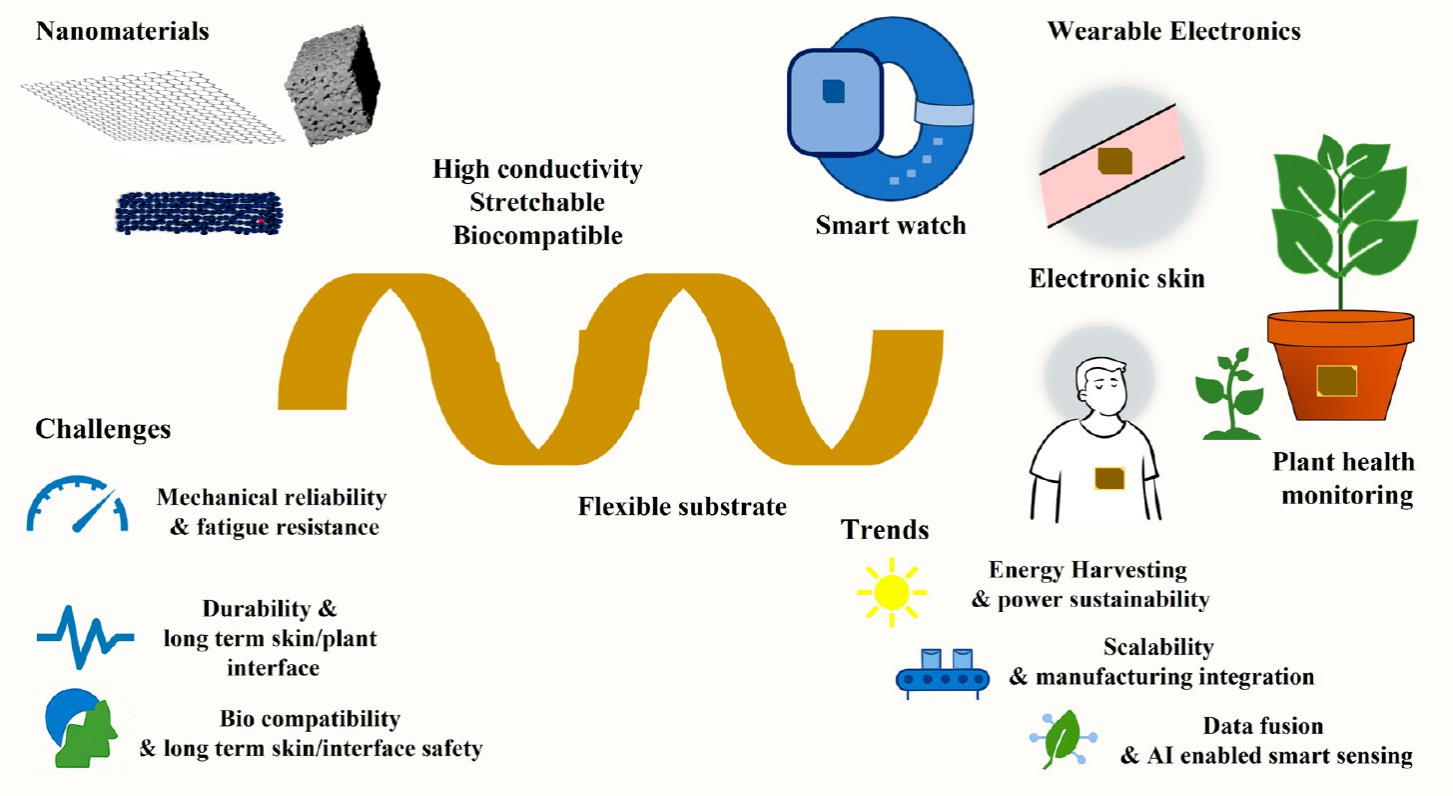
Scheme of flexible electrodes for wearable electronics from materials to integrated systems.

## 2 Fabrication of Flexible Electrode Materials

Carbon-based electrode materials are crucial in the development of flexible and wearable electronics due to their exceptional properties that meet the requirements of advanced energy storage systems. The activated carbon, graphene, and carbon nanotubes have a high specific surface area, which improves electrode–electrolyte interaction and charge storage capacity. Their high electrical conductivity enables efficient charge transmission, enabling for rapid charging and discharging, which is crucial for high-power applications in portable devices. Furthermore, the mechanical flexibility of carbon-based materials enables supercapacitors to withstand bending and stretching without losing performance, which makes them ideal for inclusion into wearable devices. Their inherent chemical stability provides long-term dependability, while their versatility allows for simple processing into a variety of forms, broadening their application. Furthermore, many compounds based on carbon, particularly those generated from biomass, are both environmentally friendly and cost-effective, enabling large-scale manufacturing while adhering to environmental sustainability objectives. These combined advantages make carbon-based electrodes important for advances in flexible, wearable, and portable electronic applications.^14^ With the rapid advancements in wearable electronics, electronic textile industries, and medical simulation, flexible electrodes are necessary to maintain the stable chemical properties of energy storage devices used in these applications, even after repeated stretching and deformation. Because of their long cycle life, mechanical flexibility, and other benefits like being economical and eco-friendly, flexible supercapacitors have attracted a lot of interest. The power density, energy density, durability, and flexibility of the flexible electrode material are all critical. Several modifying aspects are essential for charge storage capacitance in flexible electronics, including composite fabrication, the design of porous architectures with numerous dimensions for electrodes, and natural properties.^15^

### 2.1 Carbon-Based Materials

Flexible electronics require electrodes with high mechanical strength. Carbon-based materials are ideal options for flexible batteries and supercapacitors due to their outstanding mechanical characteristics. Carbon nanomaterial composites, such as graphene, activated carbon, and CNTs, are excellent candidates for flexible electronics due to their superior mechanical properties and are widely used due to their electrical conductivity, lightweight nature, and sustainability. Carbon nanostructures’ accessible surface area and ion diffusion rate can be modified to increase capacitance. Unfortunately, this material’s small surface area and poor surface electrochemical reactivity limit its use as an electrode.^16^ Shen et al. fabricated a prototype energy storage device using carbon fiber one step KOH activation (20 M KOH, 800 °C, N_2_, 30 min) followed by reheating at 800 °C in N_2_ for 30 min after washing to neutral pH with an impressive energy density of 2.58 mWh g^–1^ along with high tensile strength exceeding 1000 MPa and pressure tolerance over 100 MPa. They activated commercial carbon fibers to form nanoporous thread electrodes (Figure 2a–c) resulting in a three-order-of-magnitude increase in specific surface area while retaining 86% of the original mechanical strength. The supercapacitor fabrics are woven by solid electrolyte-coated carbon fiber threads as illustrated in Figure 2a. The device, constructed using solid electrolyte-coated threads, demonstrated excellent flexibility and mechanical stability during repeated bending tests. A photo of a load bearing supercapacitor strap with the 4-cell connect-in-series design shown in Figure 2b. Figure 2c shows a load bearing supercapacitor fabric with the size of 2.5 cm × 2.5 cm and a watchstrap made of load bearing supercapacitor fabrics powering a screen on a human wrist. As a demonstration of its practical application, a supercapacitor watchstrap was used to power a liquid crystal display, highlighting the potential for load-bearing, wearable energy storage systems with diverse form factors.^17^ Patil et al. synthesized a combination of polyaniline (PANI) and *Pisum sativum*-derived activated carbon (PSAC) was pyrolyzed at 800 °C for 2 h in N_2_ and then ball-milled for 3 h. For activation, PSC was mixed with KOH (4:1) in ethanol, dried, repyrolyzed at 800 °C for 2 h in N_2_, washed to remove residues, and dried at 80 °C for 24 h which act as an effective electrode material for supercapacitors. They achieved a maximum specific capacitance of 446 and 517 F g^–1^ on SS with specific energy of 89 and 101 W h kg^–1^, and 91% capacitance retention after 10,000 cycles at 2 A g^–1^.^18^ Koo et al. employed colorless polyimide produced from polyamidoacetic acid imidization to create graphene-flexible new transparent electrodes for organic solar cells. Monolayer graphene was grown on a 25-μm-thick copper foil via low-pressure CVD. The researchers obtained a photovoltaic conversion rate of 15.2%, resistance of just 83 Ω sq^–1^, and transmittance of light of up to 92%.^19^

**Figure 2:**
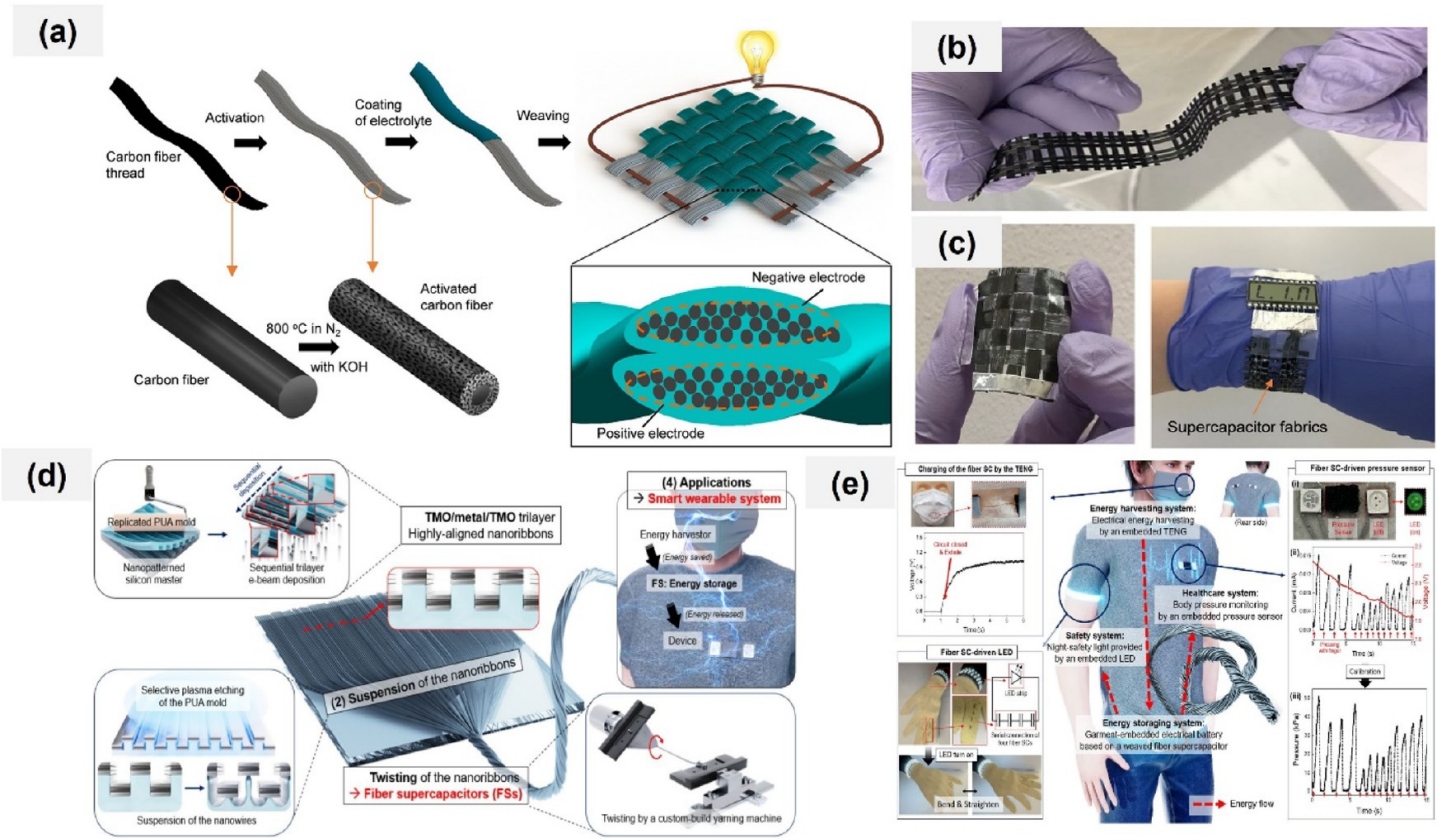
Fabrication of wearable electrode materials. (a) Fabrication process for the load-bearing supercapacitor fabrics by using activated carbon fiber thread and cross-section schematics of the supercapacitor fabrics showing two crossing threads as positive and negative electrodes. (b) A photo of a load bearing supercapacitor strap with the 4-cell connect-in-series design. (c) A photo of a load bearing supercapacitor fabric and a watchstrap made of load bearing supercapacitor fabrics powering a screen on a human wrist. Reproduced with permission from ref 17. Copyright 2022, Springer Nature. (d) Overall concept of the developed fiber supercapacitor. (e) Application of fiber supercapacitor as an energy storage component in smart textiles. Reproduced from ref 20. Copyright 2024, Wiley.

Chen et al. created flexible microsupercapacitors using the manual screen-printing platform was used to manufacture interdigital electrodes with a carbon-based composite aqueous ink containing graphene, multiwalled carbon nanotubes, and conductive carbon black. The researchers achieved an areal capacitance of 12.94 mF cm^–2^ with a current density of 0.02 mA cm^–2^ and a consistent cycling life of 102.4% capacitance retention after 10,000 cycles.^21^ Graphene oxide was prepared via the modified Hummer’s method, and heteroatom doping with boron and phosphorus in the reduced graphene oxide matrix yielded a maximum specific capacitance of 436 F/g. The electrode has a power density of 394 W kg^–1^ and an energy density of 12.48 Wh kg^–1^. It retains 97.6% after 10,000 cycles at 3 A g^–1^.^22^ Zhou and his colleagues created N/P codoped porous carbon composites with a hierarchical structure utilizing Arg­[H_2_PO_4_]_2_. The generated Arg-2–900 demonstrates a stable bilayer capacitance as a result of the quick movement of electrolyte ions in the hierarchical porous structure. The specific capacitance retention is up to 94% after 10,000 cycles.^23^ Zhao et al. designed multilayered hollow carbon spheres/CF@MoSe_2_ using hydrothermal method with carbon fiber as the substrate and transition metal sulfides to synchronize the electromagnetic properties. The absorption performance of multilayered hollow carbon spheres is highly impacted by the specific architecture of their core–shell structure.^24^

Moreover, the discussed carbon-based electrode fabrication commonly involves techniques including chemical vapor deposition (CVD), electrospinning, and pyrolysis. CVD-grown graphene and CNT films exhibit excellent conductivity and transparency, making them ideal for flexible photovoltaics; however, high-temperature growth and transfer steps increase cost and can weaken film adhesion. Electrospinning followed by controlled carbonization produces highly porous nanofiber mats with interconnected channels that are well-suited for wearable supercapacitors and strain-tolerant power systems. Biomass pyrolysis and activating agents such as KOH yield micro/mesoporous carbons with enhanced ion accessibility and environmental advantages, though mechanical strength varies with precursor microstructure. Alternatively, screen and inkjet printing of carbon-rich inks provides cost-effective large-scale patterning of electrodes directly onto fabrics, but conductivity depends strongly on the ink formulation and post-treatments. Overall, the ability to engineer porosity and surface functionality enables carbon-based electrodes to satisfy both flexibility and high-rate energy storage requirements.

### 2.2 Transition Metal Compounds

Transition metals outperform carbon materials in terms of specific capacitance and resistance. Because of their high conductivity, these materials are used as electrodes in batteries and supercapacitors to generate significant amounts of power and energy. Metal oxide improves interfacial pseudocapacitance efficacy ten to a hundredfold. Metal oxide is more energy dense and electrochemically stable than carbon or polymer materials. Hybridizing conductive carbonaceous materials like graphene, carbon nanotubes, and carbon fibers with pseudocapacitive materials results in high-performance flexible composite electrodes.^16^ The SILAR method was used to create nickel oxide thin films with three different cationic precursors, resulting in electrodes with specific capacities of 120 C g^–1^, 517 C g^–1^, and 1147 C g^–1^ at a current density of 1 mA cm^–2^. The device retained 97% of its capacitance at a 175° bending angle.^25^ Huang et al. used a vacuum filtering approach to make MnO_2_/rGO electrodes, which were then integrated into a rechargeable zinc-ion battery. The Zn-MnO_2_/rGO battery has higher capacity (332.2 mAh/g at 0.3 A/g), rate capability (172.3 mAh/g at 6 A/g), and cyclability. The capacity retention maintains 96% after 500 charge/discharge cycles.^26^ Song et al. created a multivalent vanadium oxide quantum dot-enhanced nitrogen- and sulfur-doped hierarchical porous carbon material using eggshell membrane waste biomass through hydrothermal carbonization and pyrolysis. The electrode material has a capacitance of 355 F/g at 0.5 A/g and retains 80% of its capacity at different bending angles (0°–180°).^27^ The hybrid MoS_2_-*g*-C_3_N_4_ nanocomposite electrode prepared using hydrothermal technique and demonstrates a high specific capacity of 905.0 C/g with an energy density of 25.6 Wh/kg at a power density of 844.9 W/kg, as well as 92.7% good retention and 100% Coulombic efficiency after 2000 cycles.^28^ Ahn et al. fabricated TMO nanoribbon yarns using delamination engineering of nanopatterned TMO/metal/TMO trilayer arrays, prepared through nano line molding and selective plasma etching to form twisted yarn structures. The direct formation of TMOs on Ni electrodes enabled high energy and power densities with excellent electrochemical stability in asymmetric fiber-shaped supercapacitors using CoNi_*x*_O_*y*_ yarns and graphene fibers.^20^ These FSs were further integrated with a triboelectric nanogenerator, pressure sensor, and flexible LED (Figure 2d–e). In self-powered systems, the energy-harvesting component (such as a triboelectric nanogenerator, piezoelectric generator, or photovoltaic cell) must be functionally coupled to an energy-storage unit (such as a microsupercapacitor or zinc-ion battery). This coupling is governed by several key mechanisms: (i) Charge transfer pathways, where the harvested charges are rectified and directed into the storage device through built-in rectifier circuits; (ii) Impedance matching, ensuring that the internal resistance of the energy harvester is compatible with that of the storage device to maximize energy transfer efficiency; (iii) Voltage/current regulation, where step-up converters or capacitive buffers stabilize the fluctuating output of harvesters like TENGs; and (iv) Integrated architectures, including monolithic or cable-type structures, which reduce interfacial losses and mechanical mismatch. These coupling mechanisms enable stable energy delivery and improve the operational reliability of wearable self-powered sensing platforms.

Figure 2d shows dimension-controlled pseudocapacitive TMO yarns with high electrochemical activity and rate capability are produced by twisting TMO/metal/TMO trilayer nanoribbon arrays. These nanoribbons are fabricated using industrially scalable nanoimprinting lithography (NIL) followed by electron-beam evaporation, enabling precise structural control and uniform layer formation. The applicability of the developed FS as an energy-storage component for smart textiles was demonstrated by integrating it with several functional electronic modules, including an energy harvester, a pressure sensor, and a flexible LED. To charge the FS, a single-electrode TENG was first constructed using an Au nanoribbon yarn. In this design, a single layer of Au nanoribbon yarn served as the positive electrode, while a polypropylene melt-blown mask acted as the negative triboelectric layer, forming the complete TENG system (Figure 2e).

This multifunctional system demonstrates great potential for smart textiles that combine energy harvesting, storage, and device powering. Zhao et al. developed a strain-insensitive multilayer, highly conductive, stretchable, composite material for a bioelectrode with therapeutically determined brittle contact materials. Fundamentally, the bioelectrode’s material arrangement is divided into an interconnecting element for electron transport and an interfacial element for electron transport. The authors demonstrate strain-sensitive sensing of a range of biomarkers and in vivo neuromodulation by integrating these bioelectrodes with several electrochemical probing approaches.^29^ Kwon et al. developed a stiffness-adjustable gallium–copper composite ink that has good electrical conductivity, bidirectional soft-stiffness convertibility, and high-resolution patterning, allowing for direct 3D printing of complicated, high-resolution TES circuits. Circuits can act as both mechanical conversion frameworks and electrical layers. The gallium–copper ratio in the composite ink is logically tuned using a low-copper approach, yielding a stiffness tuning ratio of 990 for 150 mm thick devices with good conductivity and high-resolution picturization. This research offers up prospects for wearables, consumer electronics, implantable devices, and robotics by simplifying and improving the adaptability of metal particle-based TES manufacture.^30^

Therefore, transition metal oxides, sulfides, and nitrides offer high pseudocapacitance but are intrinsically brittle, making fabrication strategies crucial for strain durability. Hydrothermal and solvothermal growth approaches enable the formation of strongly anchored nanostructures directly on textiles or carbon fibers, reducing interfacial resistance. Techniques like electrodeposition allow low-temperature processing and uniform coating on highly conductive substrates. Atomic-layer deposition (ALD) further enhances coating precision while maintaining intimate contact with deformable substrates, although it is limited by high cost and slow throughput. To prevent delamination during bending, these ceramics are frequently hybridized with CNTs or graphene to distribute mechanical stress and provide robust electron pathways. Despite substantial improvements in deformation tolerance, fatigue failure at the active–substrate interface remains a major bottleneck for long-term wearable applications.

### 2.3 Conductive Polymers

Conductive polymers are electrically conductive and have a large capacity. Additionally, they are more reasonably priced for pseudocapacitor usage than metal oxides. Most conducting polymers can be produced electrochemically by oxidizing the relevant monomer within the mixture. Pseudocapacitors are conductive polymers with outstanding intrinsic flexibility, redox-active capacitance, and conductivity.^31^ Ren et al. used conductive polymer Ti_3_C_2_T_*x*_ MXene/PEDOT:PSS and the graphene films were grown on copper foil by CVD using the standard process. The transparent flexible energy storage devices with a high areal capacitance of 3.1 mF cm^–2^, as well as flexibility and durability.^32^ Carbon quantum dots were doped with polyaniline and copper, resulting in a PANI-CQD-Cu composite coated on carbon cloth via electro polymerization method and achieved a maximum capacitance of 1070 F g^–1^. The flexible asymmetric supercapacitor activated carbon/PVA-H_2_SO_4_/PANI-CQD-Cu device has energy and power densities of 23.10 μW h cm^–2^ and 0.978 mW cm^–2^, respectively, with 92% capacitance retention after 3000 cycles.^33^ Gao et al. placed silver nanowires on cotton fabrics, and the AgNWs/cotton fibers served as a conductive substrate with good electrical conductivity for charge transfer. The PANI/AgNWs/cotton fiber electrode has the maximum specific capacity of 154 F g^–1^, and can sustain 96% after 5000 charge/discharge cycles.^34^ Wang et al. proposed a novel technique to interfacial engineering for organic–inorganic–organic composite fiber electrodes using seaweed fibers. The conformal composite fiber electrode has an impressive areal capacitance of 1025.6 mF cm^–2^ and an ultrahigh mechanical strength of 321 MPa. As-assembled symmetrical FESC device has a high energy density of 5.49 μW h cm^–2^.^35^

Thus, conductive polymers—particularly polyaniline (PANI) and PEDOT:PSS—are inherently flexible and are processed through electro polymerization, dip-coating, or interfacial polymerization. These fabrication routes promote conformal coating of polymer chains onto soft substrates, generating tight interfacial bonding and facilitating efficient charge transfer. Additionally, the polymerization conditions can be tuned to modulate nanostructure morphology, thereby improving mechanical compliance and redox-active charge storage. However, conductive polymers experience significant volumetric swelling and contraction during cycling, which can lead to structural degradation and loss of conductivity. Compositing with silver nanowires, cellulose fibers, or MXenes has proven effective in enhancing toughness, elongation, and cycling stability while retaining high pseudocapacitive activity.

### 2.4 MXene-Based Electrode Materials

MXenes, a form of transition metal nitride, carbide, and carbonitride found in flexible electronic devices, have been intensively studied. MXenes, a developing two-dimensional material, have a high potential for progress in domains such as sensors, energy conversion, and storage due to its remarkable chemical activity, electrical conductivity, mechanical strength, adsorption properties, and flexibility. The trade-off between transmittance and resistance makes it challenging to achieve exceptionally conductive and transparent MXene electrodes for flexible photodetectors, despite MXenes having emerged as one of the representative materials for flexible electrodes for electronic devices.^36,37^ Jiang et al. demonstrated a flexible and transparent MXene-AgNW hybrid electrode for fully solution-processed QLEDs. It combines solution-processed MXene flakes with a highly conductive AgNW prepared by polyol reduction method. Efficient welding of wire-to-wire junctions with MXene flakes gives an electrode with a low sheet resistance and high transparency of roughly 13.9 Ω sq^–1^. The manufactured electrode exhibits maximum external quantum and current efficiencies of 9.88% and 25.8 cd/A, respectively.^38^ Wang et al. developed a self-powered flexible humidity sensing system using a monolayer MoSe_2_ piezoelectric nanogenerator and poly (vinyl alcohol)/Ti_3_C_2_T_*x*_ electrospinning nanofiber sheets. The flexible PET substrate-based PVA/MXene nanofiber humidity sensor, powered by a monolayer MoSe_2_ PENG, has a high response of around 40, a short response/recovery time of 0.9/6.3 s, and a low hysteresis rate of 1.8%.^39^ Liang et al. devised ammonium polyphosphate cross-linking within the interlayer of Ti_3_C_2_T_*x*_ MXene sheets. The assembled supercapacitor has a maximum specific capacitance of 597.8 F/g (1777 F cm^–3^). The Ti_3_C_2_T_*x*_ MXene supercapacitor electrode prepared by vacuum drying method with N/P terminals has an energy density of 12.5 Wh kg^–1^ and a power density of 250 W kg^–1^.^40^ Waheed et al. developed a humidity sensor with 2D Ti_3_C_2_T_*x*_ MXene nanosheets as vacuum filtered electrodes and graphene oxide as a sensing layer. The sensor had rapid reaction and recovery times of 0.8 and 0.9 s, respectively, and maintained steady performance for 24 h.^41^ Wen et al. developed a bendable MXene/manganese dioxide ink was injected into a clean HP 803 ink cartridge, held stand for 3 min to balance the ink in the ink cartridge, and then loaded into the HP Deskjet 2132 printer. The MXene film has an ultrahigh volumetric capacitance of 312 F cm^–3^ and retains 130% of its initial capacitance after 5000 charge/discharge cycles. Furthermore, the manufactured flexible symmetrical supercapacitor has a better areal energy density of 0.51 μWh cm^–2^ at a power density of 12.5 μW cm^–2^.^42^ Yuan et al. developed a stretchable, biocompatible energy system that integrates wireless charging, energy storage, and a light-controlled switching circuit. Patterned partially oxidized liquid metal (o-LM) formed coils, collectors, and interconnects, while strong hydrogen bonding with MXene electrode fabricated using minimally intensive layer (MILD) method enabled high-performance, biaxially stretchable microsupercapacitors with a capacitance of 121 mF cm^–2^. A photodiode–triode circuit and fabrication process provided in Figure 3a. Figure 3b illustrates that the integrated system can undergo multiple mechanical deformations including stretching, twisting, winding, and crumpling-without exhibiting any visible damage. This robustness is attributed to the strong interfacial stability among the MXene layer, the o-LM coating, and the underlying PDMS substrate. Figure 3c shows a photograph of MSC attached to the finger joint, with wearability that integrates seamlessly with the skin and a mature wireless charging module was further introduced to design an energy supply system integrating wireless charging with energy storage devices. Furthermore, as demonstrated in Figure 3d, the tandem MSCs were able to adhere securely to the surface of a balloon and power a commercial LED after charging. Notably, the LED maintained a constant brightness even as the balloon expanded and contracted, indicating excellent mechanical adaptability of the device. The system reliably powered wearable and implantable devices, demonstrating stable energy delivery and confirmed biocompatibility. The MSC device implantation process in mice is shown in. Implanted 2 weeks later, the skin tissues of the implantation site and other major organ tissue sections Figure 3e. Electrical stimulation is widely used to restore motor function, requiring high current and a strong power supply for effective muscle contraction. Programmable output voltage is also needed for precise control. To achieve this, a triode and photodiode are added (Figure 3f) to amplify current and enable on-demand power switching.^43^

**Figure 3:**
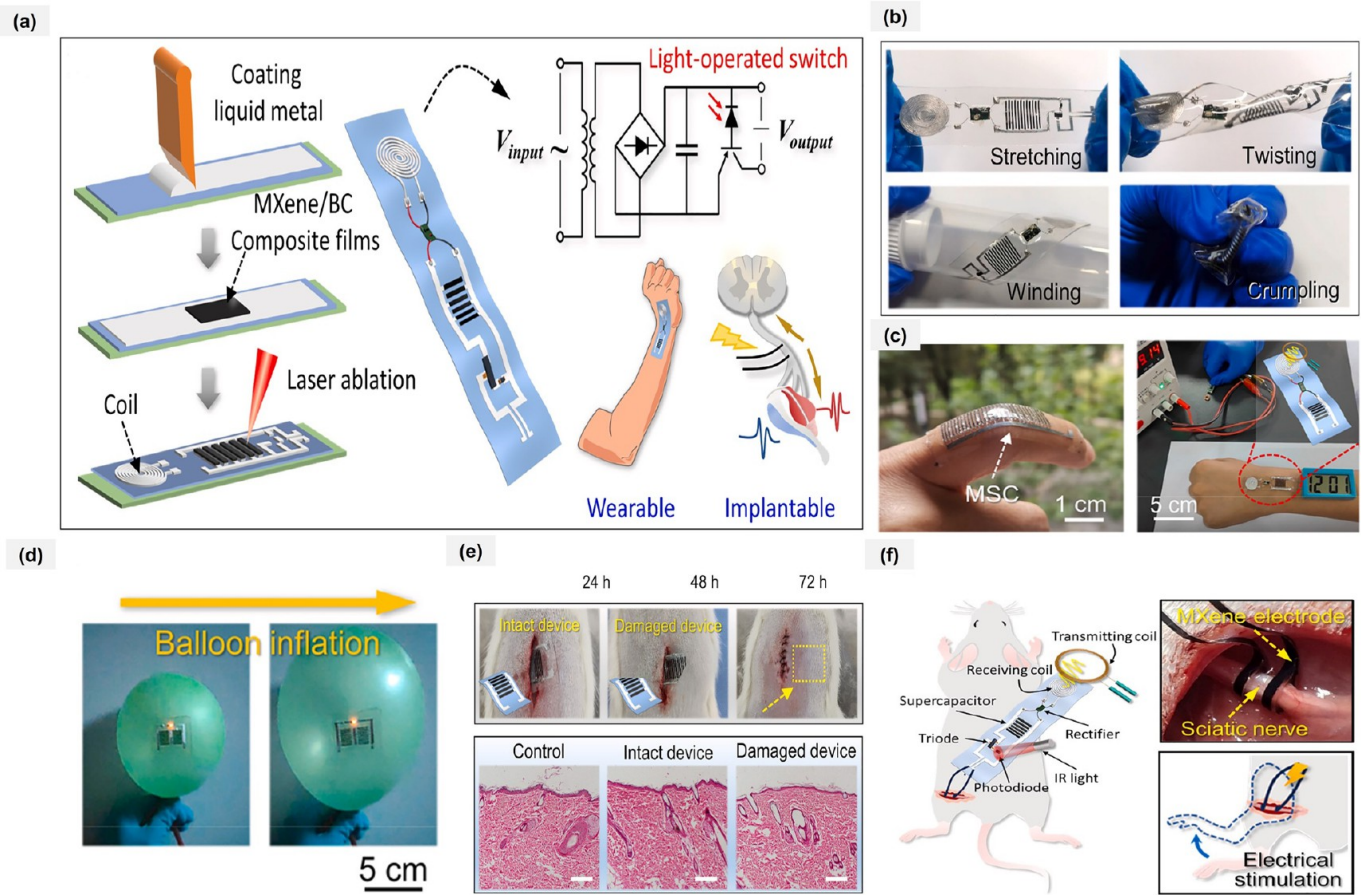
MXene based wearable electrodes and its applications. (a) The fabrication process, circuit diagram, and application scenarios of the integrated system. (b) Photographs of the system in stretched, twisted, wound, and crumpled states. (c) A photograph of MSCs attached to the finger and power supply demonstration of the system adhered to the wrist for wireless charging and powering an electronic watch. (d) Photographs of tandem MSCs array attached to an inflating balloon. (e) Photographs of the process for MSC devices implanted into the subcutaneous area and immunostaining images of skin tissues of SD rats. (f) The system applied to sciatic nerve electrical stimulation of an SD rat. Reproduced with permission from ref 43. Copyright 2025, Elsevier.

Kallupadi et al. created a triboelectric nanogenerator with polyacrylonitrile on both sides as triboelectric pairs and metal–organic frameworks (MOFs) such as ZIF-8, ZIF-67, MIL-100, and HKUST-1 via an electrospinning technique. Incorporating ZIF-8 and MIL-100 into PAN nanofibers resulted in differing triboelectric polarity trends. The pair produced an open-circuit output voltage of 100 V, a short circuit current of 1.35 μA, and a power density of 18.4 mW/m^2^, respectively. Furthermore, the gadget functions as a self-powered humidity sensor, responding quickly to changes in ambient humidity levels with a maximum sensitivity of 2.14 V/%RH. Consequently, Ti_3_C_2_T_*x*_ are typically prepared via selective etching of MAX phases to expose 2D conductive layers, followed by vacuum filtration or spray/inkjet printing to form flexible films. Their hydrophilic surfaces enable solution processing into porous membranes or stretchable electrode coatings. Integration into electrospun polymer nanofibers provides breathable textile-like devices suitable for biosensing and distributed power networks. Despite their exceptional conductivity, MXenes are prone to surface oxidation and interlayer restacking, which reduce lifespan and ion accessibility. Consequently, fabrication strategies increasingly incorporate polymeric cross-linkers or interlayer spacers to maintain high flexibility and structural stability under bending.

### 2.5 Metal–Organic Frameworks

MOFs are an important class of porous crystalline materials formed through coordination interactions between organic ligands and metal sites. They have many redox active sites, a large specific surface area, and pore diameters that may be modified. 2D-conducting MOFs (c-MOFs) have emerged as a viable choice for flexible electrode materials due to their particular structural and physicochemical properties, which include regular nanochannels for effective ion adsorption and desorption as well as strong conductivity for rapid electron transport. Currently, template approach, vacuum filtering, and hydrothermal method are the key ways commonly used to make MOF flexible electrode materials.^44^ Lu et al. developed a flexible paper electrode using Ni-MOF composites, carbon nanotubes, gold nanoparticles, and poly­(vinyl alcohol). The developed CCP electrode prepared via vacuum filtration technique can be used to identify target DNA in complex serum samples and has excellent sensing performance for HIV detection. Other pathogens’ POC can also be diagnosed utilizing the flexible, low-cost, and simple MOF composite membrane electrode.^45^ Chu et al. developed a NiCo-MOF flexible electrode for a supercapacitor. The device has a maximum specific capacitance of 1317 F g^–1^ at 1 A g^–1^ and retains 89% capacity after 15000 cycles. The completed flexible symmetric supercapacitor has an energy density of 72.55 W h kg^–1^ and a power density of 408.61 W kg^–1^.^46^ The triboelectric nanogenerator was created by employing polyacrylonitrile on both sides to form triboelectric couples with metal–organic framework using hydrothermal and solvothermal technique. The fabricated device demonstrated effectiveness for mechanical energy harvesting applications, as well as a self-powered humidity sensor, displaying rapid response to changes in ambient humidity levels with a maximum sensitivity of 2.14 V/%RH, for relative humidity range between 50 and 90% during the humidifying cycle.^47^ A summary of the key properties, limitations, and applications of these materials is presented in Table 1.

**Table 1:** Comparison of Flexible Electrode Materials

Material	Preparation methods	Strength	limitations	Applications
Carbon based (Graphene, CNTs, Activated carbon)	CVD; hydrothermal synthesis; electrospinning followed by carbonization; biomass pyrolysis/activation; screen/inkjet printing	High electrical conductivity; good mechanical flexibility; scalable fabrication routes; renewable precursors available	Limited pseudocapacitive activity; structural fragility under high strain; restacking effects	Supercapacitors; wearable electronics
Transition Metals (Oxides, Sulfides)	Hydrothermal/solvothermal growth; electrodeposition; ALD; solid-state synthesis	High capacitance and pseudocapacitive performance; good electrochemical stability	Brittle nature; complex synthesis; possible delamination during repeated bending	Batteries; hybrid supercapacitors
Conductive Polymers (PANI, PEDOT: PSS)	Electropolymerization; chemical oxidative polymerization; dip-coating; layer-by-layer assembly	Intrinsic redox activity; low cost; high flexibility; simple processing	Limited cycling stability; structural swelling/shrinkage	Pseudocapacitors; flexible sensors
MXenes (e.g., Ti_3_C_2_T_*x*_)	Selective etching of MAX phases; vacuum filtration; spray/inkjet printing; solution casting	Metallic conductivity; hydrophilicity; tunable surface terminations	Oxidation sensitivity; restacking; reduced stability in humid environments	Sensors; energy storage devices
Metal–Organic Frameworks (MOFs)	Solvothermal/hydrothermal synthesis; electrodeposition; template-assisted conversion to MOF-derived carbons	High porosity; tunable structure; multifunctional chemistry	Low intrinsic conductivity; limited moisture stability	Supercapacitors; catalytic and biosensing platforms

MOFs possess high porosity and tunable chemistry, but most pristine MOFs suffer from extremely low intrinsic electrical conductivity (typically 10^–12^ to 10^–8^ S cm^–1^), primarily due to their localized metal–ligand coordination and lack of delocalized π-electron pathways. This severely limits their direct use as electrodes in flexible supercapacitors and batteries. Recent strategies to improve MOF conductivity include: (i) forming MOF–carbon composites (with graphene or CNTs), which can increase conductivity to 10^–3^–10^–1^ S cm^–1^; (ii) incorporating redox-active ligands or metal nodes to promote charge delocalization; (iii) using conductive 2D MOFs with extended π–d conjugation; and (iv) converting MOFs into MOF-derived carbons or metal oxides to preserve porosity while achieving conductivities comparable to traditional electrode materials. These approaches significantly enhance the electrochemical performance and mechanical stability of MOF-based flexible electrodes.

MOFs are manufactured through hydrothermal or solvothermal self-assembly and can be grown directly onto soft substrates, providing large accessible surface areas for ion storage and molecular sensing. In applications requiring higher conductivity, MOFs are transformed by pyrolysis into porous metal/carbon hybrids with preserved morphology and significantly enhanced electronic transport. Vacuum filtration of MOF-based suspensions also yields lightweight, flexible membranes suitable for sensing or catalytic interfaces. While MOFs offer superior porosity and tunability, their low intrinsic conductivity and limited moisture stability continue to constrain their standalone use in flexible electrochemical devices.

### 2.6 Summary and Key Fabrication Insight

Across all material systems, the fabrication method determines how well a flexible electrode maintains electron/ion transport under repeated deformation. Carbon electrodes excel in scalability and mechanical resilience, conductive polymers in stretchability, MXenes in electrical conductivity, and transition-metal compounds in charge storage capability. However, interface engineering remains central to addressing performance decay caused by strain-induced cracking or active material detachment. Therefore, future progress in flexible electrodes will rely on fabrication strategies that integrate strong mechanical bonding, hierarchical transport networks, and scalable manufacturing compatibility.

## 3 Progress of Electrolyte Development

The electrolyte is also a key factor influencing FSC energy storage performance. Despite its capability to act as medium for high ionic conductivity electrolytes, water has a breakdown voltage of 1.23 V. This significantly limits the increase in the energy density/power density of the FSC. Furthermore, the usage of liquid electrolytes can readily leak, causing significant harm to both the equipment and the environment. Flexible polymer gel electrolytes are the best solution for improving FSC wearability and practicality. Gel electrolytes are currently classified into four types (ionic liquid gel polymer electrolyte, redox gel electrolyte, aqueous gel polymer electrolytes and nonaqueous gel electrolytes). Polymer gel electrolytes are prepared in one of two ways such as chemically or physically. Physical cross-linking techniques such as hydrogen bonding, chain connections, and electrostatic interactions are commonly employed to create polymer gels. Also, the most popular physical ways for creating polymer gels include spinning, blending, and freeze–thaw. On the other hand, chemical approaches can be divided into three categories (polymer cross-linking, graft copolymerization, and monomer polymerization) all of which use chemical linkages to form three-dimensional network gels.^48^

### 3.1 The Aqueous Gel Polymer Electrolyte

Poly­(vinyl alcohol), an organic polymer-acid gel electrolyte, bends exceptionally well and is affordable, simple to produce, and easy to create films with. Gong et al. developed a gel-type electrolyte made of NaClO_4_ and PVA. The constructed electrode has a maximum voltage window of 1.8 V and a stable temperature range of −25 to 80 °C, even after repeated bending. It retains an energy density of 37 Wh kg^–1^ and 92% of initial capacitance at ambient temperature following three cooling/heating cycles across a temperature range of over 100 °C.^49^ Zheng et al. created a free-standing flexible Agar and PAM membrane that was swollen with 1 M ZnSO_4_/2 M Li_2_SO_4_ aqueous electrolyte in air for 12 h to create ultrastable flexible aqueous lithium–zinc hybrid ion batteries. The entire battery (Zn/AP-HGPE/LiMn_2_O_4_) retains 80% of its initial capacity after 500 cycles at 1 C.^50^ Meng et al. a self-healing hydrogel electrolyte composed of zinc ions, chitosan, and polyacrylamide (Zn^2+^-CHI-PAAm) for wearable electronics and achieved a maximum capacitance of 31 mF cm^–2^.^51^ Wang et al. created a gel polymer electrolyte with physically cross-linked dopamine grafted sodium alginate and a hydrophobic association network to suppress Zn dendrite formation. The electrolyte has a cycling lifespan of over 2000 h at 2.0 mA cm^–2^ for flexible zinc-ion batteries.^52^ Saifi et al. fabricated a 90 μm-thick flexible energy harvesting and storage system (FEHSS) that integrates high-performance organic photovoltaics with zinc-ion batteries in an ultraflexible design. Figure 4a shows the photograph showing the same FEHSS attached to a textile. Scale bar: 1 cm. The system achieves a power conversion efficiency over 16%, power output exceeding 10 mW cm^–2^, and an energy density greater than 5.82 mWh cm^–2^, making it well-suited for powering wearable sensors and devices. Figure 4b shows ultraflexible OPV module consisting of 12-cells-in series and 14 of these groups in a parallel connection. Free from bulky and rigid components, the FEHSS offers a lightweight, adaptable power solution. Figure 4C show the on-shirt FEHSS positioned on the wearer’s shoulder to effectively harvest sunlight and power an on-skin ECG biosensor connected to a flexible PCB. The processed signals are transmitted via Bluetooth, enabling real-time waveform display on a smartphone. This innovation holds strong potential to advance wearable electronics and support sustainable energy technologies.^53^ Yang et al. created an antifreezing leather gel electrolyte made of leather fibers, Zn­(OTF)^2^ solution, and ethanol (EtOH) and reached a high specific capacity of approximately 70 mAh/g with 96% efficiency after 120 plating/stripping cycles.^54^ Zhang et al. fabricated a nucleotide-tackified adhesive organohydrogel electrolyte comprised with DMAEMA, AM, AMP, gelatin, and LiCl which was dissolved in the water and glycerol binary solvent. The integrated organohydrogel-based supercapacitors exhibit a specific capacitance of 163.6 mF cm^–2^ with capacitance retention of 90.6% after 5000 charging/discharging cycles at −20 °C.^55^ Zhu et al. fabricated silver (Ag)-zinc (Zn) fibrous batteries with outstanding cycle performance via designing a bifunctional gel electrolyte. By inhibiting both the migration of Ag and the formation of Zn dendrites, the graphene oxide added to the electrolyte improves structural integrity and prolongs cycle life. With a current density of 0.5 mA cm^–1^, the fabricated fibrous battery has a high capacity of 0.85 mAh cm^–1^ and can maintain 90% of its initial capacity after 250 cycles.^56^

**Figure 4:**
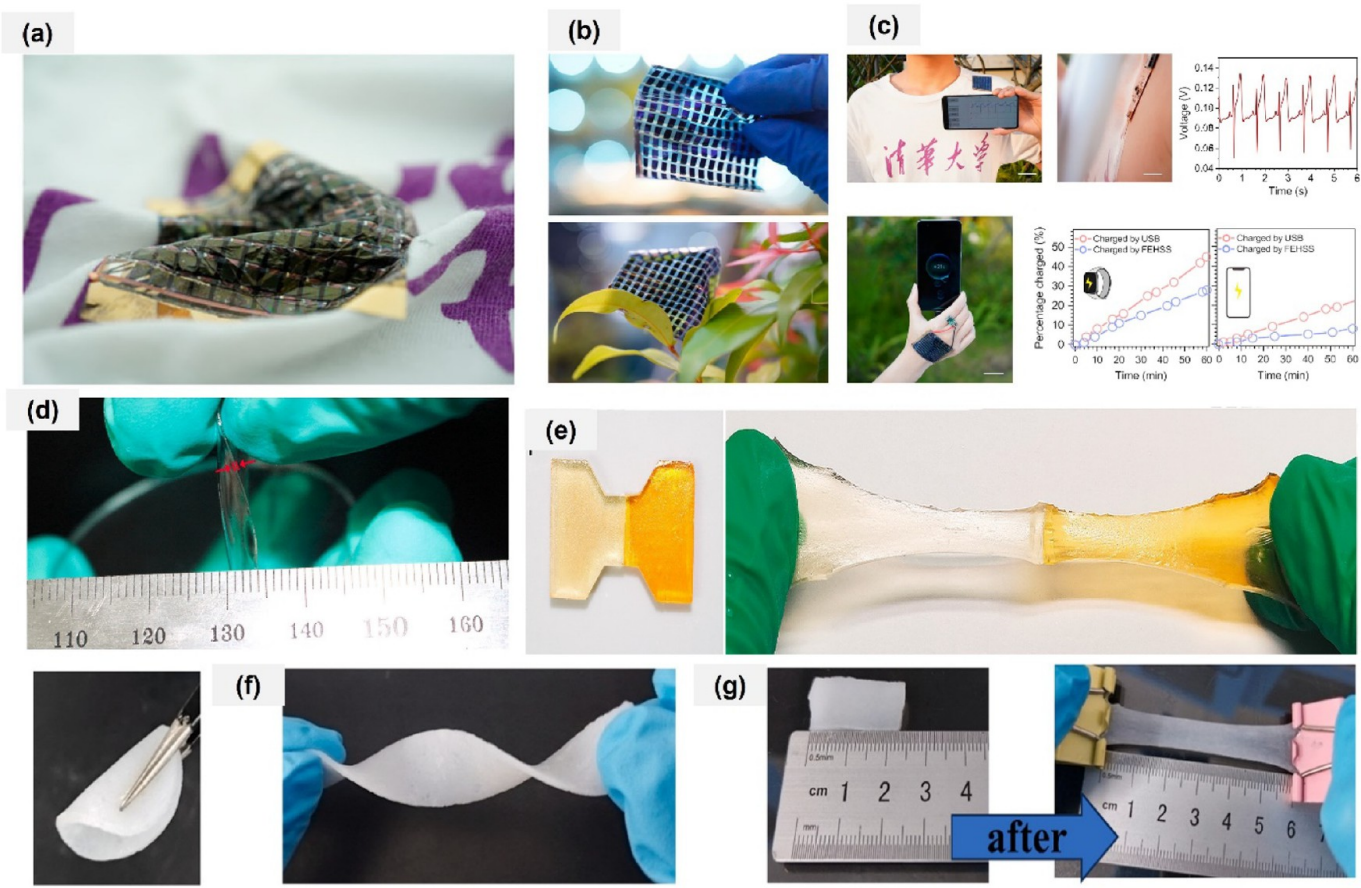
Fabrication of self-healing and stretchable polymer-based gel electrolytes. (a) Photograph showing the same flexible energy harvesting storage system attached to a textile. (b) A Photographs showing an ultraflexible OPV module. (c) Photographs of an all-flexible ECG sensing system powered by our FEHSS and Photograph demonstrating an on skin FEHSS charging a smartphone outdoors under a light intensity of 65 mW cm^–2^. Reproduced with permission from ref 53. Copyright 2024, Springer Nature. (d, e) Photographs of the healed gel in initial state and after stretching. Reproduced with permission from ref 57. Copyright 2024, Elsevier. (f, g) Optical photograph of foldable, bendable properties of PC–PVA/Zn (CF_3_SO_3_)_2_ gel electrolytes Reproduced with permission from ref 58. Copyright 2025, Elsevier.

### 3.2 Nonaqueous Ionic-Based Gel Polymer Electrolyte

Nowadays, organic compounds comprise the vast majority of electrolytes. However, organic solvents are dangerous and harmful to the environment, and improving their conductivity is difficult. They are also volatile. Ionic liquid-based gel electrolytes provide several advantages over aqueous and organic electrolytes, including large working potential windows, incombustibility, nonvolatile and high ionic conductivity. Ionic liquids have numerous applications, including functioning as liquid electrolytes, electrolyte salts in organic solvents, and improving the performance of solid polymer electrolytes.^59^ Wen et al. created a supercapacitor with poly­(methyl methacrylate-1-vinyl-3-ethyl-imidazolium bis­(trifluoromethylsulfonyl)­imide). At room temperature, it has the highest ionic conductivity among polymer electrolytes, reaching up to 2.09 × 10^–3^ S cm^–1^. It also has the highest specific capacitance, power density, and energy density, reaching 219 F g^–1^, 1907 W kg^–1^, and 115 Wh kg^–1^.^60^ Jiang et al. used an ionic electrolyte of 1-ethyl-3-methylimidazolium tetrafluoroborate to create supercapacitors with a high energy density of 28 Wh kg^–1^ and power density of 18 kW kg^–1^, retaining 83% capacitance even after 9000 cycles.^61^ Dong et al. developed gel polymer electrolyte membranes, poly­(TTT-PEMP)/G4-LiTFSI, with ion conductivity close to 10^–3^ S cm^–1^ at room temperature and better electrochemical stability for lithium-ion battery storage.^62^ Chang et al. created a polymerizable DES using anhydrous diallyl dimethylammonium chloride and urea. Figure 4d shows the photograph of PDES gel film with a thickness of 140 μm. The DES has a high transparency of 94%, a wide range of temperature tolerance of −40∼100 °C, outstanding elastic properties (ε > 2400%), good conductivity (0.05 mS·cm^–1^), and self-healing capability. The two separated gel were spliced together, and then irradiated by ultraviolet light, the unpolymerized monomer cross-linked, and the rich hydrogen bond interaction also began to reform at the fracture, which could withstand tension without fracture was shown in Figure 4e. The immaculate gel film was successfully attached to motion sensors, while the DES gel containing lignin was used as an elastic anti-UV film. This polymerizable DES can not only accelerate the creation of electronic devices such as motion sensors.^57^

### 3.3 Redox Gel Electrolyte

Recently, a new avenue in the electrolyte sector was discovered which is utilizing redox additives to increase the performance of energy storage devices.^63^ Redox gel electrolytes are soft, flexible electrolytes that combine a polymer matrix with redox-active species to enhance both ionic conductivity and energy storage performance. Typically composed of polymers like PVA or PEG, these gels incorporate redox couples such as hydroquinone/benzoquinone, iodide/triiodide, or ferro/ferricyanide, along with ionic salts (e.g., KOH or Na_2_SO_4_) to facilitate ion transport. Unlike conventional gel electrolytes, redox gels contribute additional faradaic (redox) charge storage, significantly boosting capacitance and energy density. Their flexible, nonvolatile nature makes them ideal for wearable and stretchable energy devices, although challenges like redox species stability and diffusion limitations must be managed for long-term performance. Hu et al. developed a new redox-active, stretchable, and double-network hydrogel electrolyte 4-hydroxy-2,2,6,6-tetramethylpiperidine-1-oxy-containing polyacrylamide/alginate/H_2_SO_4_ (PAM/SA/H_2_SO_4_/TEMPOL). The supercapacitor has a high specific capacitance (386 F g^–1^) and energy density of 24 Wh kg^–1^.^64^ Tu et al. developed a redox-active poly­(vinyl alcohol)­(PVA)/lithium sulfate (Li_2_SO_4_) and 1-butyl-3-methylimidazolium iodide (BMIMI) gel polymer electrolyte for a foldable supercapacitor. The constructed gadget has a high energy density of 29 Wh kg^–1^.^65^ Qin et al. created a redox-mediated, physically cross-linked double network poly­(acrylic acid)/polyisodecyl methacrylate/K_3_[Fe­(CN)_6_] gel polymer electrolyte for a supercapacitor. The supercapacitor has high self-healing and frost resistance at −10 °C, with energy density of 271 mF/cm^2^ and 98 μWh/cm^2^.^66^ Xiong et al. developed aqueous zinc–iodine batteries with a unique water reducer-based gel electrolyte, PC–PVA/Zn­(CF_3_SO_3_)_2_, resulting in a Zn anode with a good lifespan over 4000 cycles at 1 mA cm^–2^ and an average Coulombic efficiency of 99%. As shown in Figure 4f and g, the PC–PVA (10%)/Zn­(CF_3_SO_3_)_2_ gel electrolyte can be freely folded and bent, demonstrating excellent flexibility. Moreover, the gel can be stretched to nearly three times its original length without fracturing (Figure 2g), confirming its strong tensile properties. The built Zn||I_2_ complete cells have a reversible capacity of 155.2 mAh g^–1^ after 5000 cycles at 1 A g^–1^.^58^

## 4 Applications

The rise of wearable devices has sparked considerable public interest and has tremendous economic and cultural ramifications, resulting in changes to medical procedures and personal lifestyles. Flexible electronic technology is one of the most promising areas of wearable technology development. Flexible electronics offer advantages like as ultrathinness, mechanical flexibility, and high integration, making them suitable for various applications. Researchers have built flexible electronics with various shapes and purposes, including supercapacitors, batteries, sensors, and solar cells. Wearable electronic gadgets are enhancing people’s daily life with applications like medical care, protection, health monitoring, flexible energy supply systems, and AI. Wearable electronic devices have the primary problem of being stable and self-powered. Energy storage devices, an essential part of self-powered devices, can effectively solve the problem of changes in light intensity on solar cells caused by day/night, weather, and seasons, resulting in unsustainable and unstable power supply to the equipment. Conventional energy storage devices are unsuitable for wearable energy storage due to their weight, size, and rigidity. They also struggle to meet fast charging requirements.

Health monitoring, as a key component of modern healthcare, has grown to not only nurture a reliance on constantly updated data, but also to handle and process an expanding volume of data. The ability to collect and handle data in real time, precisely, and noninvasively has emerged as a major barrier in health monitoring. Self-powered tactile sensors may be effectively integrated into wearable devices to noninvasively monitor human health conditions such as heartbeat, blood pressure, respiration, and movement, which is crucial for long-term health maintenance and illness prevention. It also implies that ensuring that sensors reliably capture weak physiological signals is crucial to the trustworthiness of monitoring data. To give real-time, precise health feedback, efficient algorithms must be used to handle and analyze enormous amounts of data. However, delivering a consistent and stable power supply for these devices is an ongoing challenge. Traditional power supply techniques necessitate the use of built-in batteries or frequent connections to external power sources for recharging, which adds weight and size to the item in issue while also limiting the design’s flexibility and continuous energy supply. By transforming the biomechanical energy created by human limb movement, cardiopulmonary activity, and blood flow into electrical energy, it lowers the need for standard batteries, enhances convenience, and allows for longer monitoring periods. Similarly, modern robots have comparable issues. With the expansion of robotics applications, there is a greater need for precise motion control, awareness, and comprehension of the surroundings in unstructured situations. Tactile feedback from these sensors allows robots to do complicated tasks more precisely and adaptably, such as fine manipulation and texture recognition. By harnessing environmental energy, this technology reduces a robot’s reliance on external power sources and improves its capacity to function autonomously in unattended areas. However, in complex working situations, the sensors must be durable and trustworthy enough to provide stable performance over time. Self-powered tactile sensors fulfill these needs with their unique capacity to detect physiological signals in real-time, providing additional impetus to the development of robotics.

### 4.1 Health Monitoring

Individuals can use health monitoring to maintain track of their health state, recognize prospective health problems, and take necessary preventative or therapeutic measures. Flexible electronics are emerging as a novel trend in health monitoring due to their self-powered capabilities, flexibility, and high sensitivity. These devices can generate electricity by capturing and converting mechanical energy (such as human movement, heartbeat, and respiration). Electronic gadgets, such as wearables and remote health monitoring systems, offer a new approach to track and assess a person’s health. Flexible energy storage systems can be used to monitor a variety of health factors, including heart rate, breathing rate, and blood pressure. Blood pressure and pulse, as crucial physiological signal markers, are useful in determining an individual’s cardiovascular health. Continuous blood pressure monitoring is an excellent means of monitoring patients with chronic hypertension and assessing the risk of events, allowing preventive interventions to be done. Sonigara et al. created a patch for integrated biosensors made of NiPS_3_/graphene zinc-ion hybrid supercapacitor material. The temperature sensor is installed on the supercapacitor, which has 86% capacity after 1000 charge–discharge cycles and excellent mechanical flexibility.^67^ Liu and Peng employed graphene as an electrode material and created nanofiber networks via laser carbonization. A network of graphene nanofibers was combined with a flexible matrix and embellished with transition metal nanoparticles to form NeuroString is a flexible sensor for monitoring monoamine neurotransmitters that may be applied to the stomach or brain without interfering with normal organ function. NeuroString can detect two chemical signals with great sensitivity while without interfering with the host’s physiological activities. Real-time brain chemical reprogramming might be achieved with implanted closed-loop technology, paving the path for innovative and powerful interfaces between the brain and computers.^68^ To examine a wide range of clinical symptoms produced by PAD, Xin et al. developed an ultralight, thin biophotonic sensor capable of detecting many essential variables for continuous cardiac monitoring. The findings were strongly linked to the results of ABI and CTA investigations, and the sensor precisely, directly, and quickly reflected tissue hemodynamic characteristics. Individuals with low lesion surfaces and hypertension indicated that the sensor could correct ABI erroneous findings. The skin photoelectric biosensor has significant promise for research into sports medicine, organ tissue oxygen mediation, and AI PAD monitoring. Figure 5a presents the conceptual schematic of the on-skin biosensing system, which integrates miniaturized optoelectronics, serpentine-honeycomb interconnects, a colorless PI substrate, and a biocompatible package with a wireless module. As shown in Figure 5b, three GaAs-based LEDs (660, 750, 850 nm) and two silicon photodetectors positioned 10 and 20 mm away form a multichannel optical sensing structure for spatially resolved light absorption. A 150 nm Au/Cr layer patterned into a serpentine-honeycomb mesh provides low-resistance, biaxially stretchable electrical interconnects (Figure 5c). This network is supported by an ultrathin (∼2 μm) transparent PI film and encapsulated using biocompatible medical-grade adhesives (Figure 5d–g). Owing to its ultrathin, low-modulus design, the system conforms naturally to skin and accommodates routine deformation without restricting movement. Flexible electrodes used in medicine are frequently composed of biocompatible materials and may easily penetrate the skin, muscles, and even brain regions. This decreases injury to the human body while increasing user safety by eliminating the need for frequent bending and folding during surgery. Human forearm venous occlusion was performed to simulate limb insufficiency by placing an inflatable cuff around the biceps to block venous-but not arterial-blood flow, with the biosensor positioned on the forearm (Figure 5h). Arteriography showed blockage of the plantar arch and posterior tibial arteries (Figure 5i), explaining the false-positive ABI; after treatment, rSO_2_ returned to normal and the ulcer healed, matching CTA results. In subject 10, even though the SFA was reopened, ABI and rSO_2_ stayed low and symptoms improved only slightly because microcirculation remained poor. This shows that the biosensor can reveal persistent tissue ischemia even when large arteries appear treated, helping guide further clinical decisions. The cuff pressure was set to 100 mmHg for 3 min, during which the arm gradually turned purplish-red and the rSO_2_ steadily declined (Figure 5j). Upon release, the device detected a rapid rebound and overshoot in rSO_2_ before returning to baseline. Although absolute signal levels vary by body location, the ratios and thus oxygenation accuracy remain unaffected. Good adhesion also lowers noise interference and signal loss, hence improving signal quality. However, there are several disadvantages when compared to rigid electrodes. Low signal repeatability and stability, easily influenced by external elements such as body temperature, blood pressure, and heartbeat, rendering the signal worthless. Then implantation of the human body is costly and difficult, requiring specific processes and methods that require for a high level of medical skill and implantation equipment.^69^

**Figure 5:**
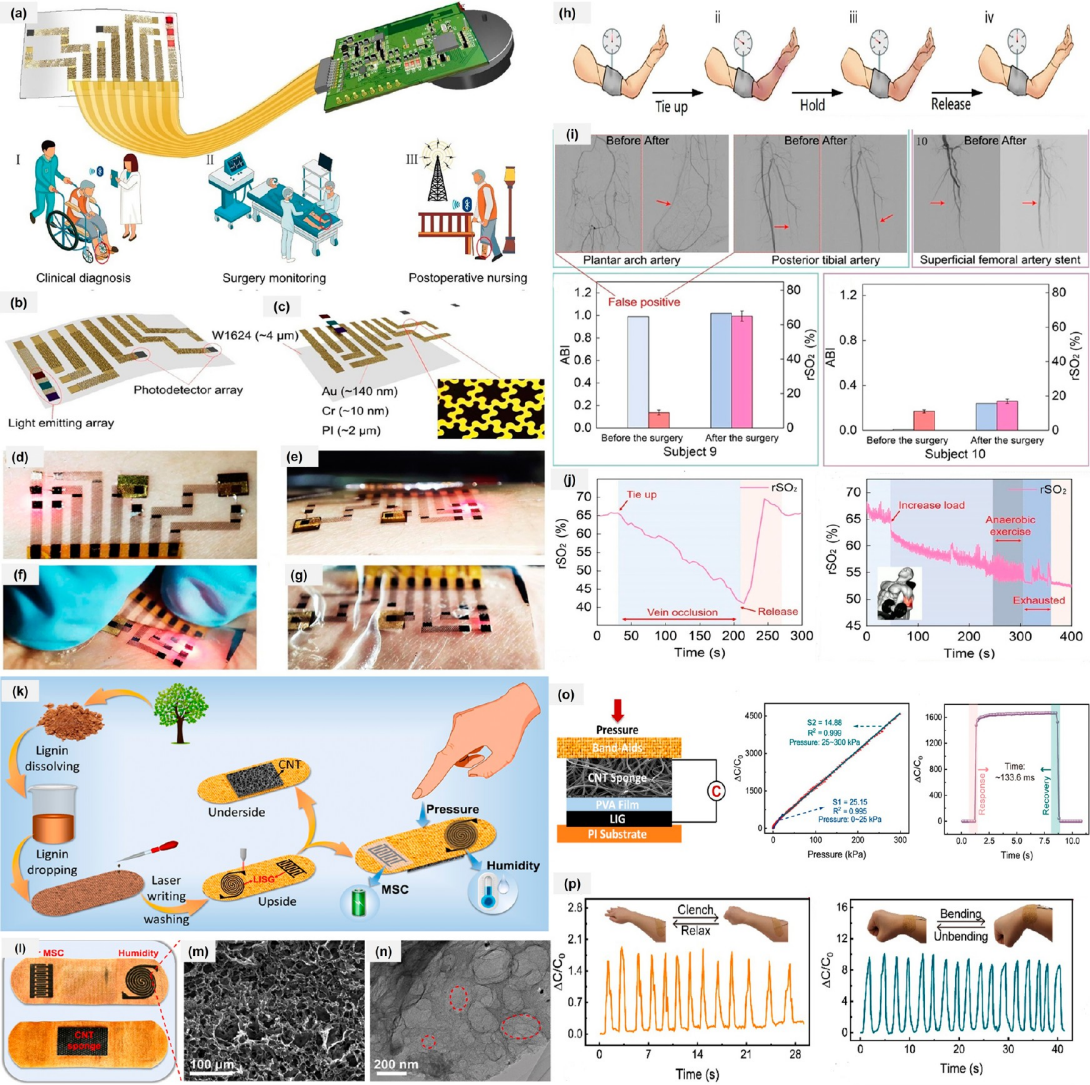
Fabrication and wearable electrodes applications in sensor and energy storage. (a) Schematic diagram of the device integrated with a flexible circuit for monitoring regional tissue hemodynamic (clinical diagnosis, surgery monitoring and postoperative nursing. (b) Front view and (c) exploded view of the on-skin integrated photoelectric element, the near-infrared LED sensor. (d) Image of the biosensor placed on the skin. (e–g) The device is superconformal and deformed by extrusion. LED, light-emitting diodes; PD, photodetector. (h) The process before, during, and immediately after venous occlusion. (i) Functional demonstration during transient vein occlusion (blue). (j) A weight-lifting exercise was performed for the biceps brachii, and the change in muscle oxygen saturation was monitored during the exercise; moreover, the critical point when the muscle changed from aerobic to anaerobic respiration was determined. Reproduced with permission from ref 53. Copyright 2023, Springer Nature. (k) Schematic diagram of the multifunctional band-aid and the preparation processes of the LISG electrodes. (l) Digital photos of LISG electrodes with interdigital and spiral patterns on the band-aid surface and the CNT sponge electrode on the backside of the band-aid. (m) SEM image and (n) TEM image of the LISG sample. (o) Schematic diagrams of the capacitive pressure sensor and Relative capacitance variations, the response and relaxation time when loading and unloading onto the pressure sensor and (p) Relative capacitance response to various human activities of arm muscle contraction and wrist bending. Reproduced with permission from ref 72 .Copyright 2023, Elsevier.

Overall, flexible health-monitoring devices offer excellent conformability, high sensitivity, and real-time physiological tracking compared with rigid sensors, but they still face challenges such as motion-induced artifacts, long-term biocompatibility concerns, and the need for reliable wireless communication during daily activity.

### 4.2 Flexible Sensor and Energy Storage

Flexible batteries, noted for their flexibility, shock resilience, and lightweight design, have great promise for use in building and wearable electronics. As demand rises, traditional hard batteries may gradually decline. While certain solutions allow for electrode flexibility, today’s flexible batteries frequently have low specific capacity and operate poorly. This has resulted in the creation of three-dimensional electrode structures, such as arrays, linear, and porous supports. Flexible supercapacitors, with their great flexibility, quick charging capabilities, and long-lasting cycling stability, are potential alternatives. FSCs commonly employ two- and three-dimensional electrode architectures to improve electrochemical and mechanical properties via material selection and structural optimization. Additionally, flexible pressure sensors with high flexibility, sensitivity, resolution, and response speed are required for wearable electronic devices. These sensors, which are classified as piezoresistive, capacitive, and piezoelectric, have made tremendous advances, particularly in flexible, curved-surface applications, and are becoming increasingly crucial in wearable technologies and intelligent robotics. Sweat was used as the electrolyte, and the active electrode was poly­(3,4-ethylenedioxythiophene): poly­(styrenesulfonate) (PEDOT: PSS). With PEDOT: PSS coated onto cellulose/polyester cloth; the SC demonstrates specific capacitance of 8.94 F g^–1^ (10 mF cm^–2^) at 1 mV s^–1^. The SC has energy and power densities of 1.36 Wh kg^–1^ and 329.70 W kg^–1^ at 1.31 V, with a specific capacitance of 5.65 F g^–1^. The same electrode was tested with real human sweat, yielding energy and power densities of 0.25 Wh kg^–1^ and 30.62 W kg^–1^, respectively. SC performance is examined using various sweat volumes (20, 50, and 100 μL), bending radii (10, 15, 20 mm), charging/discharging stability (4000 cycles), and washability.^70^ Luo et al. created a “sweat-chargeable” on-skin SC employing a biocompatible NaCl electrolyte and freestanding PANI/CNT electrodes. Sweat from the human body can cause a redox reaction between the additional Zn foil and oxygen in the air, resulting in the device self-charging to 0.652 V in 6 min. To illustrate its wearability in real-world applications, a sweat-chargeable band is constructed that can spontaneously power a timer or pedometer when the user is exercising.^71^ Yuan et al. created a multipurpose intelligent wearable band-aid using a simple laser-scribing and single-wall carbon nanotube drop-casting approach. The laser-induced sulfur-doped porous graphene MSC on the band-aid surface has a high areal capacitance of 68.6 mF cm^–2^ and good cyclic stability. Figure 5k shows the schematic for fabricating multiple sensors on a smart band-aid, which integrates microsupercapacitors, humidity sensing, and pressure sensing. The device consists of two functional parts designed to achieve these capabilities. The LISG interdigital electrodes are utilized as MSCs, whereas the GO-coated LISG spiral electrodes operate as humidity sensors (Figure 5l). The SEM image in Figure 5m reveals that the LISG electrode possesses a rough surface with a well-defined reticular porous network structure. The TEM image in Figure 5n shows that LISG contains abundant mesopores and macropores, further confirming its porous structure. To form the MSC electrodes, a sodium lignosulfonate (SLS) slurry was first coated onto the band-aid and then rapidly laser-scribed to produce S-doped porous graphene (LISG) patterns. Figure 5o shows that the capacitive pressure sensor achieves high sensitivity, measuring 25.15 kPa^–1^ below 25 and 14.88 kPa^–1^ in the 25–300 kPa range, indicating a broad and stable linear response. Repeated measurements produced very small error bars, demonstrating low measurement error and excellent repeatability. In addition, the humidity sensor is designed to respond quickly and reversibly in real time, with a wide relative humidity detection range (11–97% RH). As shown in Figure 5p, the pressure sensor attached to the forearm can detect subtle muscle contractions during hand clenching and relaxing. Similarly, when fixed to a joint such as the wrist, bending induces pressure on the sensor, causing a measurable increase in capacitance corresponding to the bending angle. The capacitive pressure sensor has a high sensitivity of 25.15 kPa^–1^, an ultrabroad linear range (0–300 kPa), and a low limit of detection of 2.92 Pa.^72^ Yarn-based hydrogel flexible supercapacitor wrapped in a layer of fluffy and soft sensitive film constructed of reduced graphene oxide-modified cotton fibers to serve both energy storage and sensing functions. A mechanically durable, flexible all-hydrogel, sodium dodecyl sulfate-modified poly­(vinyl alcohol)@copoly­(acrylate-aniline) (PVA@P­(AA-ANi)-SDS) yarn is developed through the synergy of dynamic cross-linking and SDS-induced emulsification creation followed by freezing-copolymerization. The PVA@P­(AA-ANi)-SDS yarn electrode has a high specific capacitance of 618 F/g and an energy density of 37.3 Wh/kg. Then, a fluffy and soft sensitive rGCF film was wrapped over the surface of the yarn-based SC to create an all-yarn SC sensor. Despite severe mechanical movement and pressure, it maintains strong mechanical stability, resulting in outstanding sensitivity (25.75 kPa^–1^) and a large pressure detection range of up to 44 kPa.^73^ Zhao et al. suggested a fully woven 3D fabric electrode with silk glue and graphene oxide coatings and a flexible electrode substrate made of conductive and water-retaining composite yarn. The yarn’s water-retaining capabilities and fluffy structure can help both long-term and motion status monitoring applications by increasing electrode contact, ensuring good skin contact, and improving signal quality. Because knitted electrodes are permeable and offer a gentle touch, their porous structure improves user comfort. Electrocardiogram and electromyography signals have been experimentally and definitely analyzed.^74^ Liu et al. developed a breath monitoring and posture recognition system using hydrogel electrolytes derived from poly­(vinyl alcohol), sodium alginate, and starch, which is suited for supercapacitors and multimodal wearable sensors. They developed an artificial neural network to obtain a finger-pressing posture identification accuracy of up to 99%, and the hydrogel sensors were also successfully used in the diagnosis of obstructive sleep apnea syndrome.^75^ Wang et al. developed a new conductive aqueous glue for a thick, flexible, and strong cathode/anode by weaving carbon nanotubes into cellulose nanosheets. With the active material, the adhesive’s ultrathin 2D reticulated nanosheet structure exhibits a revolutionary “point-to-point” bonding mechanism. The flexible cell with electrodes had a high specific capacity (141 mA h g^–1^) and ultrahigh surface capacity (12.1 mA h cm^–2^). This cellulose-based glue technology works well with advanced high-performance functional devices, particularly flexible and high-energy batteries.^76^ Performance metrics of representative systems are compared in Table 2.

**Table 2:** Comparison of Flexible Electrodes and Sensors

Material System	Key Performance Metrics	Application Focus	Reference
Activated carbon fiber threads	Energy density 2.58 mWh g^–1^, tensile strength >1000 MPa	Wearable supercapacitor strap	^17^
PANI + biomass-derived activated carbon	Capacitance ∼ 517 F g^–1^, 91% retention after 10,000 cycles	Supercapacitors	^18^
Graphene + polyimide transparent electrodes	PV conversion efficiency 15.2%, Rs = 83 Ω sq^–1^	Flexible solar cells	^77^
Graphene/CNT/carbon black ink	Areal capacitance 12.9 mF cm^–2^, >10,000 cycles	Flexible microsupercapacitors	^21^
MnO_2_/rGO in Zn-ion battery	332.2 mAh g^–1^, 96% retention after 500 cycles	Rechargeable Zn-ion batteries	^26^
MXene/PEDOT: PSS	Areal capacitance 3.1 mF cm^–2^, transparent and durable	Transparent supercapacitors	^32^
MXene microgels cross-linked with polyphosphate	Capacitance 597.8 F g^–1^, energy density 12.5 Wh kg^–1^	Flexible supercapacitors	^40^
NiCo-MOF composites	1317 F g^–1^, 89% retention after 15,000 cycles	Flexible supercapacitors	^46^

Flexible sensors and energy-storage units provide the unique advantage of mechanical durability and seamless integration with deformable surfaces, yet limitations remain in energy density, long-term cycling stability, and large-scale manufacturing compatibility.

### 4.3 Robotics

Flexible electronics is an important way for humans to experience the world, and offering robots human-like tactile sensitivity is one of the essential trends in future robot development. The use of self-powered supercapacitors in robots is a cutting-edge research topic. Flexible electronics in robotics serve an important role in assisting machines to perceive and interact with their surroundings, providing robots with tactile sense while lowering reliance on external power sources. Shinde et al. created an MXene/MnCo_2_O_4_ nanocomposite supercapacitor electrode on a copper substrate utilizing a simple and inexpensive electrodeposition process. The MXene/MnCo_2_O_4_ electrode has a specific capacitance of 668 F g^–1^, a high energy density of 35 Wh kg^–1^, and outstanding cycling stability (94.6% retention after 5000 cycles). An asymmetric supercapacitor device using MXene/MnCo_2_O_4_ (positive electrode) and Bi_2_O_3_ (negative electrode) exhibits exceptional performance in powering small robotics and electronics.^78^ Nie et al. developed a soft, stretchable thermal protective substrate for wearable electronics, offering excellent thermal insulation, mechanical compliance, and stretchability. The design combines polydimethylsiloxane with embedded heat-absorbing microspheres made of phase change materials encapsulated in a resin shell. Figure 6a illustrates the design of the thermal protective substrate (TPS), which provides thermal insulation, mechanical compliance, and stretchability for skin-mounted wearable devices. The TPS is formed by embedding heat-absorbing microspheres-containing n-eicosane encapsulated in a melamine-formaldehyde shell-into a PDMS matrix to impart thermal insulation. During operation, excess heat from the device is absorbed through the solid–liquid phase change of n-eicosane, preventing overheating of the skin. The mechanical compliance and stretchability of TPS with 80 wt % heat-absorbing microspheres. Both experimental and numerical results show the substrate can endure over 150% strain and reduce peak skin temperature rise by more than 82% with optimization. In vivo tests on mouse skin further demonstrate its exceptional thermal protection for wearable thermal management applications. Depilated mice were used to evaluate the thermal protection of the TPS and after the experiment, severe blisters appeared on all mice treated with PDMS, whereas only slight blistering was observed in the TPS group (Figure 6b).^79^ Nardekar et al. created a microsupercapacitor that is extremely flexible, lightweight, and wirelessly rechargeable, making it a perfect power source for microscopic soft robots. The durable graphene-like carbon microsupercapacitor (GLC-MSC) electrode is created using a simple and scalable direct laser combustion process. The GLC-MSC has high areal capacitance (8.76 mF cm^–2^) and retains its capacity even at high actuation frequencies (1–30 Hz). The authors create a completely integrated magnetic-soft robot with exceptional mobility aptitude and charge wirelessly (up to 2.4 V within 25s), making it an ideal onboard power source for soft robotics.^80^ Yang et al. used atomic-layer deposition to create an ultrathin Al_2_O_3_ coating layer that significantly improved the electrochemical performance of vanadium diselenide nanosheets @N-CNFs (Al_2_O_3_@VSe_2_ NSs@N-CNFs) film cathodes in flexible quasi-solid-state aqueous zinc-ion batteries. Furthermore, the ALD-Al_2_O_3_ nanocoating improves mechanical resilience in the cross-2D nanosheet direction of VSe_2_ on the N-CNF surface. The device has a high energy density (125 Wh kg^–1^), ultrahigh rate capability of 65% capacity retention after 200-fold growth, and 86% retention after 2500 cycles. The quasi-solid-state aqueous zinc-ion batteries are then integrated into a soft inchworm robot, allowing for reversible actuation on both land and water while maintaining good electrochemical stability. An ultraflexible, lightweight, and wirelessly rechargeable microsupercapacitor was developed and used to power miniature soft robots.^81^

**Figure 6:**
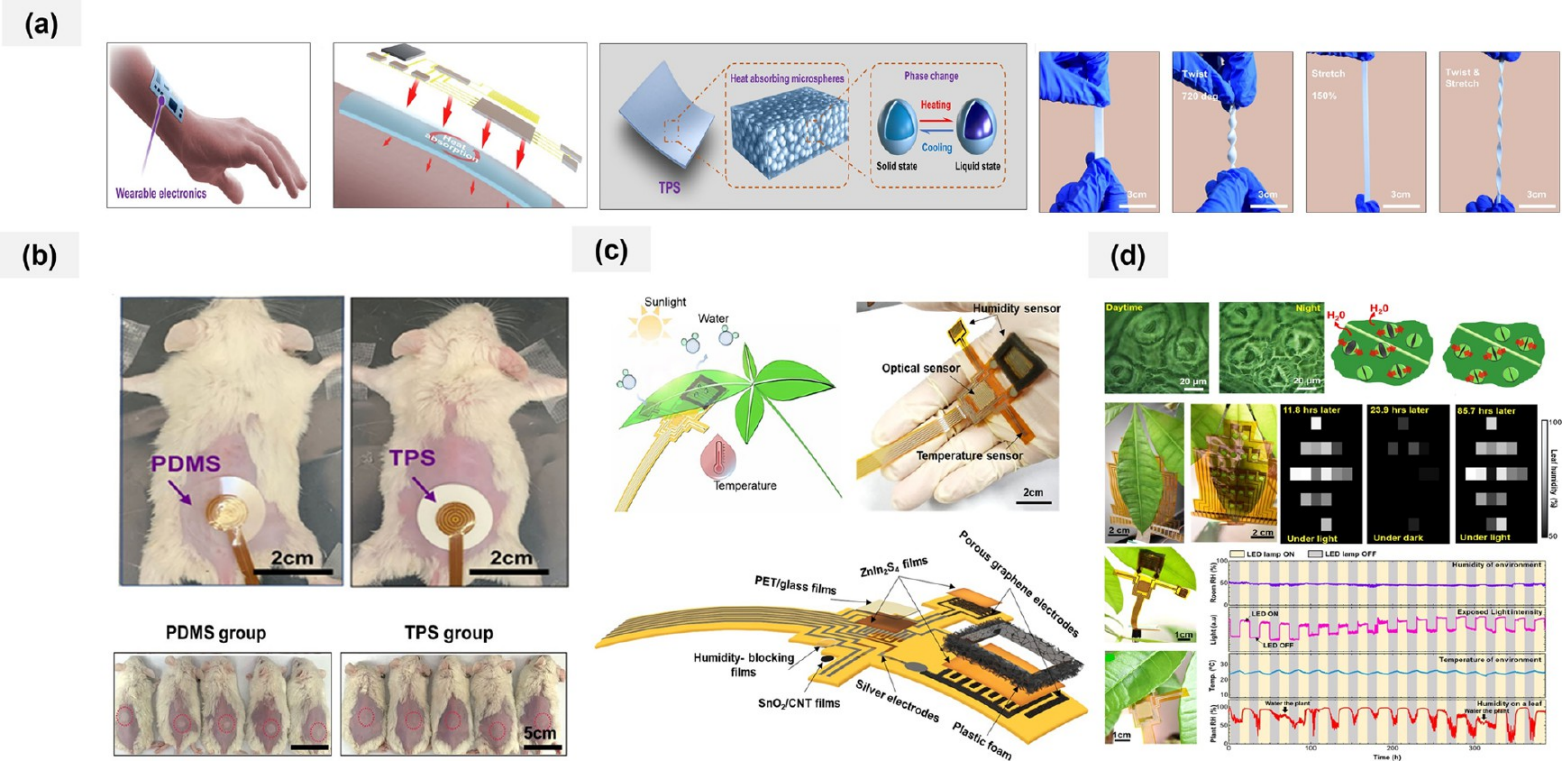
Applications of wearable electrodes in robotics, animal and plant health monitoring. (a) Schematic illustration of a wearable electronic device attached to human skin and heat transfer from devices to skin with the TPS absorbing large amounts of heat energy and TPS composed of a polymer matrix embedded by heat-absorbing microspheres consisting of phase change materials encapsulated by the resin shell and optical images of TPS under various deformations. (b) Photos of wearable heaters with the PDMS substrate (left) and TPS (right) attached to the mice skin and Photos of mice after experimenting in PDMS (middle), and TPS (right) group. Reproduced with permission from ref 79. Copyright 2022, Springer Nature. (c) Schematic of multimodal flexible plant healthcare sensors. (d) Demonstration of plant healthcare monitoring. Optical microscope images of leaf stomatal switching conditions at daytime and night with Photos of front- and back-sides of the leaf transpiration mapping device attached on the lower epidermis of a leaf with transpiration monitoring results using a multimodal flexible sensor device under room humidity, light, room temperature, and leaf humidity results are shown from the top to bottom. Reproduced with permission from ref 82. Copyright 2017 Springer Nature.

In soft robotic systems, flexible electronics enable natural motion tracking, high responsiveness, and safe human interaction, but their performance can still be hindered by material fatigue, limited output power, and complex interface requirements for high-precision control.

### 4.4 Wearables for Plant Monitoring

Flexible sensors have various advantages over traditional rigid electronics, including long-term monitoring, comfortable contact, accurate measurement, and greater biocompatibility. This section will discuss plant wearables from four different perspectives: plant growth microenvironment detection, crop quality detection, wearable-based active plant growth control, and plant physiological state detection. Oren et al. developed a tape-based wearable sensor with a patterned graphene sheet resistance of 0.22 ± 0.12 kΩ sq^–1^. A 4.4% tensile strain was achieved after loading and discharging 100 times. They successfully analyzed the timeframes required for water transfer within the maize from the roots to target leaves through the xylem by measuring variations in leaf relative humidity, which helped to explain the corn’s transit time.^82^ Wearable plant humidity sensors can help deliver more reliable and user-friendly plant breeding tools and indicators. Lu et al. developed a multipurpose flexible sensor for monitoring plant health and sensing leaf moisture using stacked ZnIn_2_S_4_ (ZIS) nanosheets. To assess plant health, a multimodal flexible sensor system is designed to monitor transpiration and estimate internal water status while simultaneously measuring ambient humidity, light, and temperature—three key abiotic factors governing plant transpiration (Figure 6c). The system enables real-time tracking of both plant physiological signals and environmental conditions. It integrates two humidity sensors, an optical sensor, and a temperature sensor into a single flexible platform for comprehensive plant-growth monitoring. To assess plant development behavior, they successfully detected changes in stomatal closure, environmental humidity, light, and temperature by integrating two humidity sensors, an optical sensor, and a temperature sensor on the flexible PI substrate. Transpiration plays a crucial role in revealing the health status of plants, as the opening and closing of stomata regulated by guard cells control the exchange of water vapor with the atmosphere Figure 6d. To assess stomatal behavior and distribution, large-area humidity mapping was performed. As shown in Figure 6d, the maps capture distinct transpiration patterns under light and dark conditions.^83^ To monitor NH_3_ levels around plants, Li et al. developed a flexible sensor made of a hybrid polyaniline/Ti_3_C_2_tx sensitive film. This sensor additionally examines how temperature and humidity influence the measurement of gases.^84^ Zhao et al. developed a wearable plant biosensor for the surface monitoring of organophosphorus pesticides. On an uneven agricultural region, a three-electrode serpentine with tensile characteristics and deformation was formed using laser induction. Methyl parathion can be selectively caught by a smart electrochemical sensor, which can wirelessly and instantaneously transfer collected data to a smartphone. This new type of plant wearable biosensor provides a novel way for in situ analysis of pesticide residues in crops and can be employed in the future development of precision agriculture.^85^ Different sensor materials and their applications are outlined in Table 3.

**Table 3:** Comparison Table for Plant Health Monitoring Wearables

Sensor Type/Material	Key Findings	Application	Reference
Laser-induced graphene humidity sensor	Real-time tracking of plant transpiration at biointerface	Plant transpiration monitoring	^86^
ZnIn_2_S_4_ nanosheet-based multimodal sensor	Monitored stomatal closure, humidity, light, and temperature	plant growth/health sensing	^83^
PANI/Ti_3_C_2_Tx hybrid film	NH_3_ detection, evaluated humidity/temperature influence	gas sensing in crops	^84^
Serpentine laser-induced biosensor	Pesticide detection (methyl parathion), wireless data transfer	precision agriculture	^85^

Plant-wearable flexible sensors allow nondestructive, continuous monitoring of plant physiology and environmental stress, offering advantages over traditional agricultural tools; however, their deployment is often constrained by outdoor environmental variability, long-term adhesion issues, and power-supply limitations.

## 5 Outlook

In summary, although significant progress has been made in developing flexible electrodes, sensors, and energy-storage systems for wearable and plant-integrated electronics, several key challenges remain unresolved. Current technologies continue to face mechanical limitations such as interfacial stress mismatch, fracture of brittle active layers, and fatigue under repeated bending, as well as chemical issues including oxidation, moisture sensitivity, and long-term environmental instability. Practical deployment is further constrained by limited energy density, signal drift during motion, and the lack of scalable, low-cost fabrication techniques. Looking forward, future developments are expected to focus on self-healing and damage-tolerant materials, stress-buffering geometries, hybrid multifunctional systems, and advanced encapsulation strategies that enhance durability under real-world operating conditions. The integration of autonomous energy harvesting, machine-learning-assisted signal interpretation, and sustainable or biodegradable substrates will accelerate progress toward robust, intelligent, and environmentally adaptive flexible electronic platforms.

## 6 Challenges

Although flexible electrodes and electrolytes have demonstrated remarkable progress in recent years, several critical challenges remain before these systems can be fully implemented in next-generation wearable electronics. First, mechanical robustness and interfacial stability continue to be major limiting factors. Many high-performance electrode materials-such as transition-metal oxides, MXenes, and conductive polymers-exhibit intrinsic brittleness or undergo structural fatigue during repeated deformation. Your reviewed studies (e.g., fiber-shaped supercapacitors, MXene–PDMS composites, MOF-derived electrodes) show that cracking, delamination, and interfacial debonding under bending, twisting, or stretching are among the key reasons for performance degradation. Addressing these challenges requires deeper understanding of strain distribution, interfacial stress mismatch, and cyclic mechanical fatigue across multilayered architectures.

Second, chemical and environmental instability remains a major barrier to long-term operation. MXenes oxidize readily under ambient humidity; conductive polymers undergo swelling/shrinkage; and MOFs display poor moisture resistance due to fragile coordination bonds. These limitations hinder the practicality of wearable devices intended for sweat-rich, outdoor, or skin-contact environments. Advanced encapsulation layers, hydrophobic surface modifications, and oxidation-resistant chemistries are needed to improve environmental reliability without compromising flexibility or breathability.

Third, electrolyte engineering is increasingly important yet insufficiently mature. Although your manuscript discusses solid, gel, and polymer electrolytes, challenges remain in achieving simultaneously high ionic conductivity, stretchability, mechanical integrity, and biocompatibility. Many gel systems dry out over time, while polymer electrolytes require trade-offs between modulus and conductivity. Future electrolytes must be engineered with ionic liquids, zwitterionic systems, deep eutectic solvents, and self-healing ion channels to overcome these limitations.

Fourth, integration of energy harvesting and storage remains underdeveloped. While your review cites TENG-powered and PV-powered systems, practical implementation requires proper impedance matching, stable rectification and power management circuits, and codesign of harvesting + storage interfaces. Fully integrated, monolithically fabricated, self-powered systems are still rare, and this represents a key direction for future wearable platforms.

Fifth, scalability and manufacturability continue to hinder commercialization. Many of the reviewed fabrication methods—such as hydrothermal growth, e-beam evaporation, or nanolithography-are not compatible with large-area, roll-to-roll, or textile-integrated production. Future research must pursue printing technologies, fiber spinning, solution-based coating, and reactive ink formulations, enabling cost-effective industrial deployment.

Finally, future development will benefit greatly from advances in research methodologies. Multiscale mechanical modeling can predict fracture points in stretchable electrodes; molecular simulations can guide electrolyte ion transport; machine learning can accelerate materials discovery; and digital-twin simulations can optimize self-powered system architectures prior to fabrication. These innovations will shorten development cycles and lead to more durable, multifunctional flexible systems.

## 7 Conclusions

This minireview provides a comprehensive synthesis of recent advances in flexible electrodes and electrolytes for wearable electronics, covering material classes such as carbon-based materials, transition-metal compounds, MXenes, conductive polymers, and MOFs, as well as a broad range of applications including health monitoring, soft robotics, energy storage, and plant physiological sensing. The reviewed studies demonstrate significant progress toward achieving high electrical performance, mechanical adaptability, multimodal sensing, and integration with self-powered platforms. Despite these advancements, substantial work remains to translate laboratory-scale demonstrations into robust, real-world technologies. Future progress will require addressing material brittleness, interfacial delamination, environmental sensitivity, limited ionic conductivity, and scaling challenges. At the system level, next-generation devices must adopt holistic design strategies, combining flexible electrodes, advanced electrolytes, efficient energy harvesters, and intelligent signal processing into seamless platforms. Continued innovations in materials chemistry, mechanical engineering, encapsulation science, and data-driven design will pave the way for new generations of highly durable, multifunctional, and fully self-powered wearable electronic systems capable of supporting healthcare, human–machine interfaces, environmental sensing, and beyond.
